# Imaging Studies of the Stifle Joint in *Puma concolor* (Linnaeus, 1771)

**DOI:** 10.3390/vetsci12020103

**Published:** 2025-02-01

**Authors:** Eduardo Burgarelli Mayrink Cardoso, Sheila Canevese Rahal, Jeana Pereira da Silva, Maria Jaqueline Mamprim, Jair Theodoro Filho, Gabriela Montezel Frigerio, Paulo Hilário Nascimento Saldiva, Mário Manuel Dinis Ginja, Karin Werther

**Affiliations:** 1 Department of Veterinary Surgery and Animal Reproduction, School of Veterinary Medicine and Animal Science, São Paulo State University (UNESP), Botucatu 18618-681, Brazil; eduardo.burgarelli@unesp.br (E.B.M.C.); jeana.silva@unesp.br (J.P.d.S.); jaqueline.mamprim@unesp.br (M.J.M.); 2 Imaging Platform in the Autopsy Room (PISA), Faculty of Medicine, University of São Paulo, São Paulo 01246-903, Brazil; jair.theodoro@hc.fm.usp.br (J.T.F.); gabriela.montezel@hc.fm.usp.br (G.M.F.); pepino@usp.br (P.H.N.S.); 3 Department of Veterinary Science, University of Trás-os-Montes and Alto Douro, 5001-801 Vila Real, Portugal; 4 School of Agricultural and Veterinarian Sciences, São Paulo State University (UNESP), Jaboticabal 14884-900, Brazil; karin.werther@unesp.br

**Keywords:** radiography, computed tomography, magnetic resonance imaging, anatomy, wild feline

## Abstract

Although the stifle joint of wild felines shares several characteristics observed in domestic cats, other characteristics are specific to each species. This study aimed to evaluate the stifle joints of eight *Puma concolor*, including two young and six adults, through different imaging examinations. All stifles were assessed using radiographs and computed tomography (CT). Magnetic resonance imaging (MRI) was performed on the stifles of one animal using 7 Tesla equipment. In all imaging modalities, the four sesamoid bones were detected. Meniscal mineralization was identified in the stifles of three adult animals and one young animal. The cruciate ligaments and menisci were identified on CT, with MRI providing better visualization. The mean values of CT measurements (cm^2^) in the sagittal section included patella (2.475), medial fabella (0.481), lateral fabella (0.772), popliteal sesamoid (0.222), and medial meniscus (0.051). No differences were found in HU values between the central trabecular bone of the patella and popliteal sesamoid, the cortical bone of the patella and lateral and medial fabellas, or the cortical bone of the patella and popliteal sesamoid. In conclusion, the descriptions of the stifle of *Puma concolor* from the different imaging methods contribute to understanding the species and can serve as a basis for identifying alterations.

## Simple Summary

Pumas are known for their agility and can jump to heights exceeding 5 m. The stifle joint is considered a complex joint, both anatomically and functionally. Although the stifle joint of wild felines shares several characteristics observed in domestic cats, other characteristics are specific to each species. Therefore, this study aimed to evaluate the stifle joints of pumas (*Puma concolor*) using digital radiography, computed tomography (CT), and magnetic resonance imaging (MRI). Imaging methods complemented each other in assessing the components of the puma’s stifle joint, as certain structures like the cruciate ligaments and meniscus were not visible on plain radiographs. The meniscus and cruciate ligaments were visualized on CT and MRI, but ultra-high-field MRI (7 Tesla) allowed these structures to be observed with precision. In the stifle joints where meniscal mineralization was present, identified in three adults and one young animal, it was detected across all imaging modalities.

## 1. Introduction

The puma (*Puma concolor*) is a mammal of the felid family, with 32 subspecies classified into six phylogeographic groups based on genomic analysis [1,2,3]. Among these groups, *Puma concolor capricornensis*, *Puma concolor concolor*, *Puma concolor cabrerae*, and *Puma concolor puma* are found in South America; *Puma concolor cougar* is found in North America; and *Puma concolor costaricensis* is found in Central America [1]. The puma is considered the second-largest feline in the Americas and the largest of the puma genus [4], and can be found from Canada to the south of South America, excluding some regions of Chile and the Caribbean islands [5,6]. This feline inhabits tropical and subtropical humid forests, temperate forests, mountainous areas, and swamps, as well as arid or cold regions, demonstrating its ability to adapt to various environments, including those near agricultural and anthropized areas [4,6].

The body mass of pumas ranges from 22 to 74 kg, with females being smaller than males [5]. Both males and females are solitary and are active during crepuscular and nocturnal hours [4,5,7]. Pumas are known for their agility and can jump to heights exceeding 5 m [5]. Sexual maturity is reached after 24 months and their lifespan typically ranges from 8 to 10 years, although they can live up to 13 years [3].

The species is classified as least concern globally by the International Union for Conservation of Nature (IUCN) Red List [2]. In Brazil, pumas are found in all biomes but face threats such as habitat loss and fragmentation due to agricultural expansion, roadkill, persecution for predation, and fires [3,5,6].

The stifle joint of domestic felines is considered a complex joint, both anatomically and functionally, consisting of the medial and lateral femorotibial, and femoropatellar joints, which form three communicating compartments [8,9]. Although the stifle joint of wild felines shares several characteristics observed in domestic cats, other characteristics are specific to each species, such as mineralization of the medial meniscus and the presence of one or two fabellas [10]. Some anatomical, radiological, and histological studies have been carried out on the stifle joint of *Puma concolor* [10,11,12]. However, there is a lack of evaluations utilizing advanced imaging techniques such as computed tomography (CT) and magnetic resonance imaging (MRI), which can avoid osseous or soft tissue superimposition, allow for cross-sectional imaging, and provide better visualization of the joint structures.

Therefore, this study aimed to evaluate the stifle joints in pumas (*Puma concolor*) using digital radiography, CT, and MRI. Additionally, CT measurements and Hounsfield Unit (HU) values were obtained to assess sesamoids and medial meniscus mineralization in the sagittal view. The hypothesis was that a combination of at least two imaging modalities is necessary for a more comprehensive assessment of the puma stifle joints.

## 2. Materials and Methods

### 2.1. Animal Selection

The methodology used in the present study was approved by the Institutional Ethics Committee for the Use of Animals (CEUA-n^o^. 0179/2022) and the National Environmental and Wildlife Bureau (SISBIO-84129-2). Hind limbs from eight pumas were used, including three males and five females, and two young and six adults, with body masses ranging from 26.5 to 51 kg (mean 41.31 kg ± 5.94). Except for one puma from a zoo, all hind limbs were obtained from roadkill animals. Since most of the animals were free-ranging, their ages were classified as young and adult. The right and left hind limbs of all animals were harvested by disarticulation at the hip joint, placed in plastic bags, numbered from 1 to 8, and stored in a −20 °C freezer for preservation until imaging exams.

### 2.2. Imaging Studies

**Figure 1:**
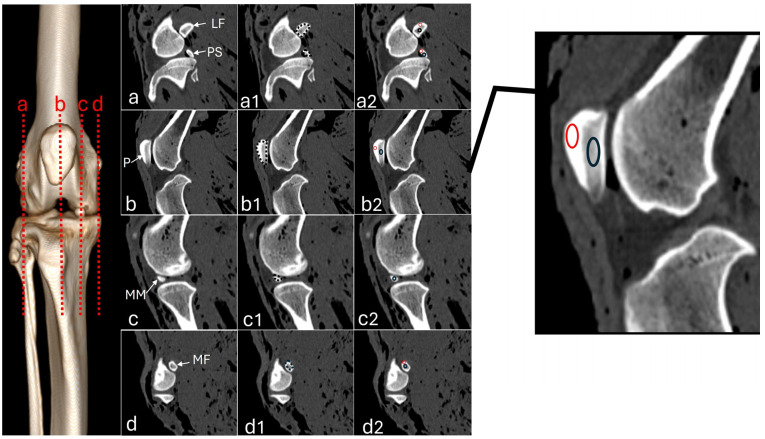
Sagittal computed tomography images of an adult puma stifle joint (*Puma concolor*). (**a**) Lateral fabella (LF) and popliteal sesamoid (PS); (**a1**) outline of the sesamoids for area measurement (dashed line), (**a2**) region of interest (ROI) of the compact bone (red circle) and trabecular area (black circle) for measuring Hounsfield Units (HU). (**b**) Patella (P), (**b1**) area measurement, (**b2**) ROIs. (**c**) Medial meniscus (MM), (**c1**) area measurement, (**c2**) ROIs. (**d**) Medial fabella (MF), (**d1**) area measurement, (**d2**) ROIs. Higher magnification to show the ROIs of the compact bone (red circle) and trabecular area (black circle).

Radiographs of the stifle joints (*n* = 16) were taken in the craniocaudal and mediolateral views with digital radiography equipment (NEOVet, Sedecal, Hefei, China). Exposure parameters were set to 60 kVp and 8 mAs, with a focus-film distance of 100 cm. CT scans were performed on a 16-channel scanner (SOMATOM Emotion, Siemens, Erlangen, Germany) with parameters set 130 kVp, 116 mA, and a 0.8 mm slice thickness. Cross-sectional images were obtained from the distal portion of the femur to the proximal portion of the tibia. Multiplanar (dorsal and sagittal) and three-dimensional (3D) reconstruction images were evaluated using RadiAnt DICOM (Digital Imaging and Communications in Medicine) Viewer software 2023.1 (64-bit) (Medixant, Poznan, Poland). In the sagittal slice, the areas of the patella, medial, and lateral fabellae (sesamoid bones located at the head of the gastrocnemius muscle), and the sesamoid of the popliteus muscle were measured (Figure 1). Additionally, Hounsfield Units (HU) were measured in each region of interest (ROI) of the sesamoids, which included one point in the compact bone area located proximally and another in the trabecular bone positioned in the center, as shown in Figure 1(a2,b2,d2). Due to the small size of the medial meniscus mineralization, the ROI was located in the central area (Figure 1(c2)). All measurements were taken by an experienced imaging veterinarian. If present, meniscal mineralization was identified based on its position in the femorotibial joint and its density was measured in HU (Figure 1).

MRI (sagittal and dorsal sections) was performed on the stifles of one animal randomly selected using 7 Tesla equipment (Magnetom 7T, Siemens Healthineers—GhMb, Erlangen, Germany). Sequences trialed for sagittal, transversal, and dorsal planes included two-dimensional (2D) T2-weighted, 3D-DESS (double echo steady-state), 3D T2 SPACE (Sampling Perfection with Application optimized Contrast using different flip angle Evolution), and 3D FLASH (fast low-angle shot).

The three imaging methods were used to identify and characterize osseous and soft tissue structures of the stifle joint.

### 2.3. Statistical Analysis

The normality of the data measurements was verified using the Kolmogorov–Smirnov test. Based on the distribution, the paired *t*-test and Wilcoxon test were used to compare the variables between the stifles and among sesamoids. A significance level of *p* < 0.05 was adopted. Statistical analyses were conducted using GraphPad Prism Version 4.0 software (San Diego, CA, USA).

## 3. Results

### 3.1. General Information and CT Measurements

Imaging exams confirmed that two animals were young because the proximal tibial and distal femur growth plates were fully open. Also, four animals had fractures: two in the left femur (nos. 6 and 7), one in the left fibula (no. 3), and one in the left fibula and both femur bones (no. 1). All fractures were pre-mortem and caused by a fatal road accident.

Table 1 displays CT measurements of the sesamoids and meniscus mineralization. The HU values for the sesamoids and meniscus mineralization in the right and left stifle joints are presented in Table 2 and Table 3, respectively.

### 3.2. Stifle Joint Description

**Figure 2:**
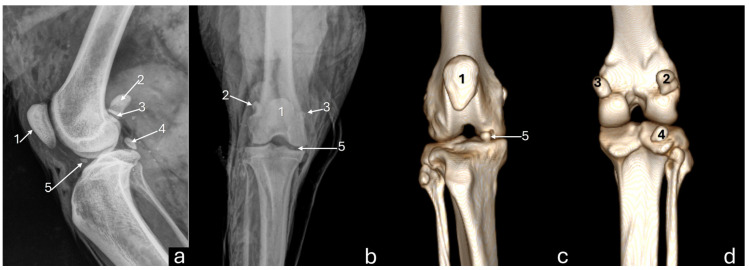
Radiographs in mediolateral (**a**) and craniocaudal (**b**) views, and 3D reconstruction computed tomography images in cranial (**c**) and caudal (**d**) views of an adult puma stifle joint (*Puma concolor*). 1—patella, 2—lateral fabella, 3—medial fabella, 4—popliteal sesamoid, 5—mineralization of the medial meniscus. Note meniscal mineralization (5) as a radiopaque structure in the medial compartment of the femorotibial joint (**a**,**b**).

On the mediolateral radiographic view (Figure 2a), the patella had a triangular shape with a wider base than the apex, positioned in the trochlear groove. The femoral condyles had a convex surface without overlap, with the lateral condyle approximately 9% larger than the medial condyle. The articular surface of the tibia had a convex appearance. The fabellae and sesamoid of the popliteal muscle were identified. An intra-articular radiopaque structure consistent with partial meniscus mineralization was observed in the stifles of three adults and one young animal. On the craniocaudal radiographic view (Figure 2b), well-defined and convex femoral condyles on the articular surface were visualized, with the lateral condyle approximately 18% larger than the medial one. The patella was oval and positioned in the trochlear groove. The extensor fossa was identified on the lateral condyle. The surface of the lateral and medial tibial condyles had a slightly convex appearance, with the lateral one being around 24% larger. The intercondylar eminence was clearly defined, showing two intercondylar tubercles, with the lateral one larger than the medial one, and a central intercondylar area. The lateral and medial fabellae were visualized as rounded radiopaque structures in the epicondylar region of the lateral and medial condyles, respectively. The lateral fabella was larger than the medial one. The head of the fibula was articulated with the tibia. In the medial compartment of the femorotibial joint, a radiopaque structure was seen, compatible with meniscal mineralization in the same four animals.

**Figure 3:**
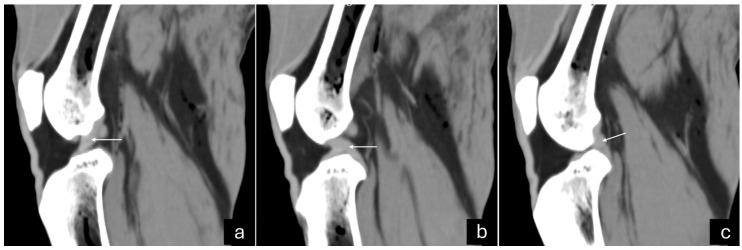
Sagittal computed tomography images on sagittal plane of an adult puma stifle joint (*Puma concolor*). (**a**) Cranial cruciate ligament (arrow) from the caudal portion of the femur to the cranial area of the tibia. (**b**) Caudal cruciate ligament (arrow) from the cranial aspect of the femur to the popliteal margin of the tibia. (**c**) Meniscofemoral ligament (arrow).

**Figure 4:**
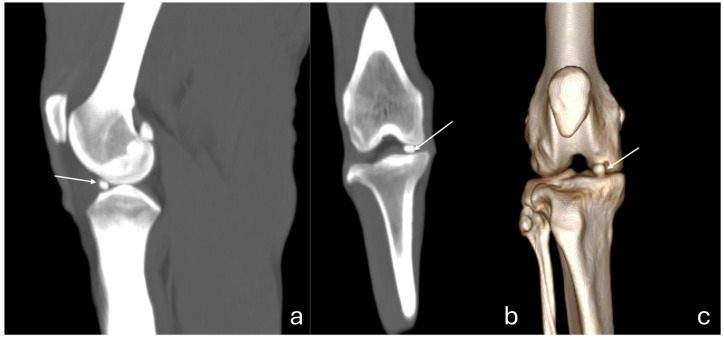
Computed tomography images of an adult puma stifle joint (*Puma concolor*). Sagittal (**a**) and dorsal planes (**b**), and a cranial view of the 3D reconstruction (**c**). Observe mineralization of the medial meniscus (arrow) as a hyperdense portion in the medial compartment (**a**,**b**).

The 3D reconstruction of CT images (Figure 2c) revealed, in a cranial view, the patella as a drop-shaped structure with a wider base than the apex, positioned in the symmetrical trochlear groove. Meniscal mineralization was identified in the stifles of the same four animals as a hyperdense portion in the medial compartment. The caudal and lateral views displayed the lateral and medial fabellae in the epicondylar region of the lateral and medial condyles, respectively, with the lateral one being larger (Figure 2d). Other bone structures showed similar patterns as seen in radiographic images. Multiplanar and cross-sectional images allowed identification of the patella, infrapatellar fat, cranial cruciate ligament (from the caudal portion of the femur to the cranial area of the tibia), caudal cruciate ligament (from the cranial aspect of the femur to the popliteal margin of the tibia), and meniscofemoral ligament (Figure 3). The menisci were more difficult to identify, but those with partial mineralization were easily visualized (Figure 4). The fabellae and sesamoid of the popliteal were also identified; all had a thin cortical layer.

**Figure 5:**
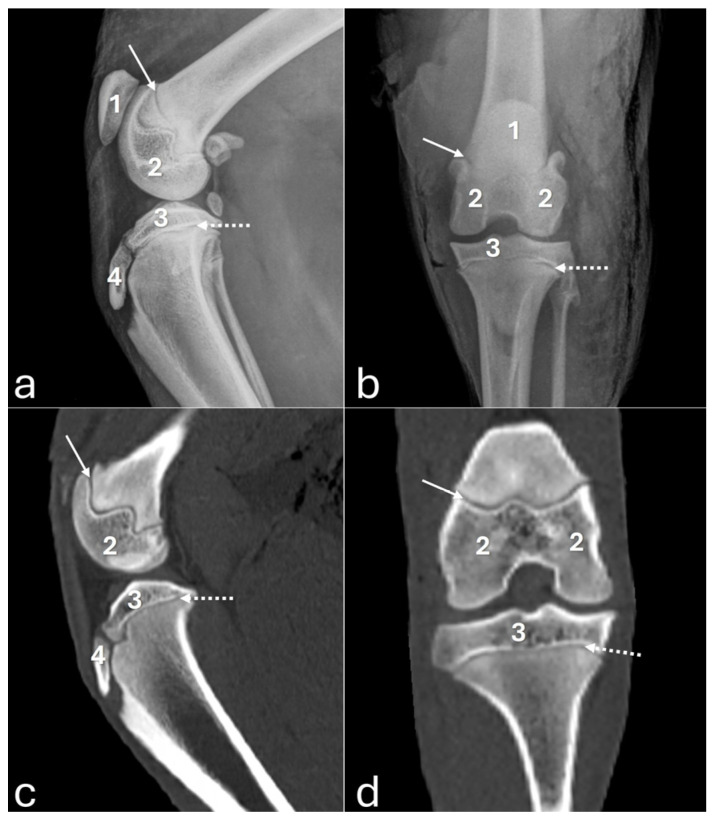
Radiographs in mediolateral (**a**) and craniocaudal (**b**) views, and computed tomography images on sagittal (**c**) and dorsal (**d**) planes of a young puma stifle joint (*Puma concolor*). 1—patella, 2—femoral condyles, 3—proximal tibial epiphysis, 4—tibial tuberosity. Arrow—distal femoral physis. Dashed arrow—proximal tibial physis. Observe all growth plates fully open.

Figure 5 displays radiographs and CT images of the stifle joint of a young puma where no meniscal mineralization was detected.

No bone or cartilage lesions were found in the stifle joints on radiograph and CT images.

**Figure 6:**
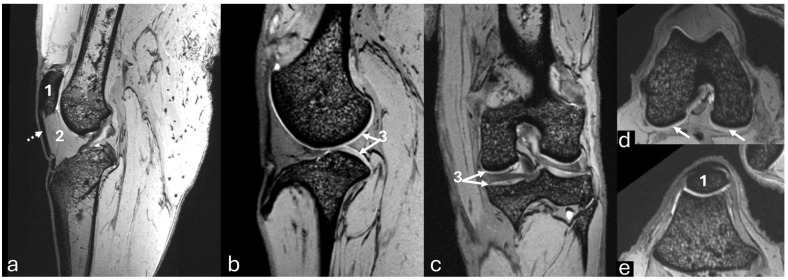
Three-dimensional double echo steady-state (3D-DESS) imaging at *7*-T MRI on sagittal (**a**,**b**), dorsal (**c**), and transversal (**d**,**e**) planes of an adult puma stifle joint (*Puma concolor*). (**a**) Observe the patella (1), infrapatellar fat pad (2), and patellar ligament (dashed arrow). (**b**,**c**) Observe the articular cartilage (white signal) on the surface of the distal femur and proximal tibia (3), and (**d**) on the femoral condyles (arrows) and patella.

The MRI images of the stifle joint structures were evaluated in all sequences, but qualitatively the 3D-DESS was considered better than the others. The 3D-DESS showed that the cartilages of the femur and tibia had a uniformly homogenous white signal in all planes and the bone contours were regular (Figure 6). High-resolution imaging of subchondral bone was also visualized. All sesamoids were identified.

**Figure 7:**
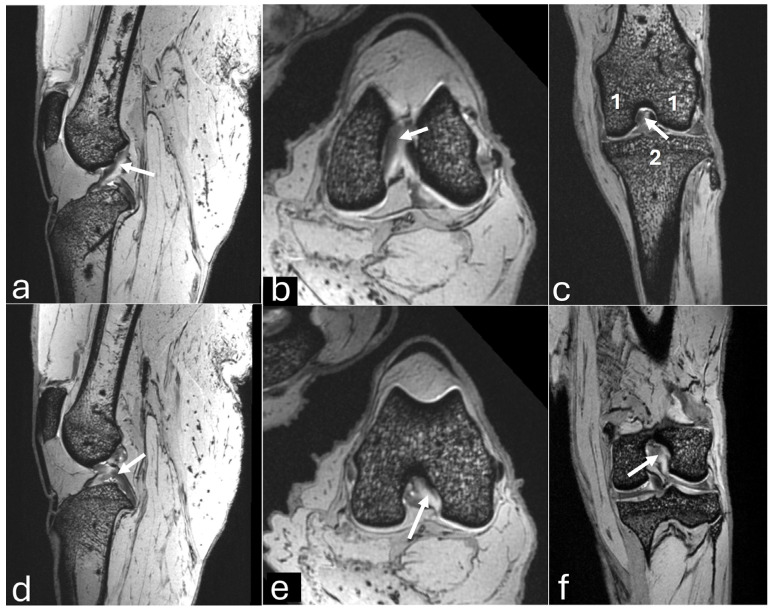
Three-dimensional double echo steady-state (3D-DESS) imaging at *7*-T MRI on sagittal (**a**,**d**), transversal (**b**,**e**), and dorsal (**c**,**f**) planes of an adult puma stifle joint (*Puma concolor*). (**a**–**c**) Observe the cranial cruciate ligament (arrow). (**d**–**f**) Note the caudal cruciate ligament (arrow). 1. Femoral condyles. 2. Proximal tibia.

**Figure 8:**
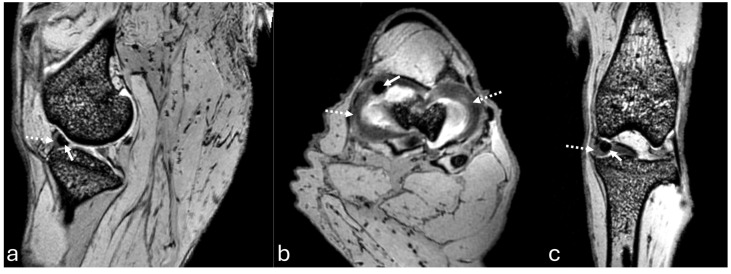
Three-dimensional double echo steady-state (3D-DESS) imaging at *7*-T MRI on sagittal (**a**), transversal (**b**), and dorsal (**c**) planes of an adult puma stifle joint (*Puma concolor*). Observe the medial meniscus (dashed arrow) and the mineralization of the cranial horn of the medial meniscus (arrow).

The sagittal plane image showed the hypointense patellar ligament beginning at the patellar apex and inserting on the tibial tuberosity, patella, patellofemoral compartment, and infrapatellar fat pad, which was located deep on the patellar ligament in the cranial part of the joint (Figure 6a). The cranial cruciate ligament ran from the caudal aspect of the femur condyle to the tibial tuberosity (Figure 7a). The caudal cruciate ligament ran from the cranial aspect of the femoral intercondyloid fossa to the popliteal notch of the tibia (Figure 7d). The meniscofemoral ligament ran from the lateral meniscus to the medial condyle of the femur. Both menisci had a hypointense signal with a triangular shape or bow-tie appearance according to the slice. The mineralization of the medial meniscus was identified in the cranial aspect (Figure 8a).

**Figure 9:**
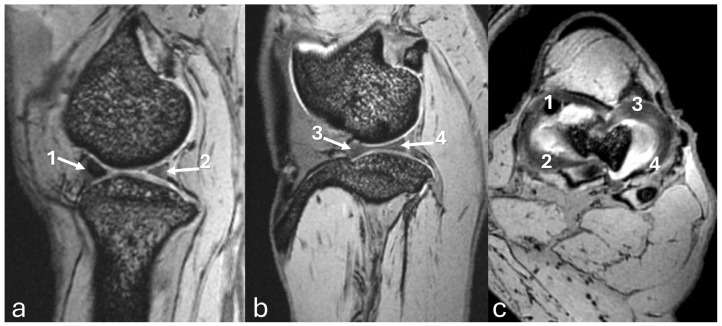
Three-dimensional double echo steady-state (3D-DESS) imaging at *7*-T MRI on sagittal (**a**,**b**), and transversal (**c**) planes of an adult puma stifle joint (*Puma concolor*). 1. Cranial horn of the medial meniscus. 2. Caudal horn of the medial meniscus. 3. Cranial horn of the lateral meniscus. 4. Caudal horn of the lateral meniscus. (**a**) Observe the C-type shape of both menisci on the transversal plane. (**a**) Note the triangle appearance of both menisci on the sagittal plane and the bow-tie appearance on the other slice of the same plane.

The transverse plane showed the cruciate ligaments (Figure 7b,e) and the C-type shape of both menisci, with the medial meniscus being larger than the lateral one (Figure 8b and Figure 9c). The cranial and caudal horns were identified (Figure 9c). Meniscal mineralization was easily identified in the cranial horn of the medial meniscus as a rounded structure with a hypointense signal (Figure 8b and Figure 9c).

The dorsal plane revealed that the medial and lateral femoral condyles articulated with the tibial plateau and formed the medial femorotibial and lateral femorotibial compartments (Figure 7c). The fibula head was located on the lateral side. The lateral and medial collateral ligaments showed a hypointense signal. The cranial and caudal cruciate ligaments were clearly visible in the intercondylar notch between the medial and lateral compartments (Figure 7c,f). The caudal cruciate ligament appeared thicker than the cranial cruciate ligament. Both menisci had a triangular shape with a hypointense signal, which was less than the mineralization of the medial meniscus (Figure 8c).

### 3.3. Statistical Analysis

No statistical differences were found in the tomographic measurements (cm^2^) and HU values of the sesamoids and medial meniscus mineralization between the right and left stifles. Therefore, the values were combined and are presented as a single value in Table 4 and Table 5. HU values of the trabecular and compact bones in the medial and lateral fabellae were similar, so their values were combined for comparison with other sesamoids. Additionally, there were no significant differences in HU values between the central trabecular bone of the patella and popliteal sesamoid, the cortical bone of the patella and fabellae of the gastrocnemius, or the cortical bone of the patella and popliteal sesamoid (Table 6).

## 4. Discussion

Imaging methods complemented each other in assessing the components of the puma’s stifle joint, as certain structures like the cruciate ligaments and meniscus are not visible on plain radiographs.

The four sesamoid bones were detected in all stifle joints, i.e., the patella, the medial and lateral fabellae, and the popliteal sesamoid, as described in the domestic cat [8]. Previous studies using two [12] or three [11] cadavers of *Puma concolor* also verified all sesamoids. The patella appeared oval on craniocaudal radiographs and drop-shaped on cranial CT reconstructions, with a triangular shape on mediolateral radiographs. The appearance resembled that described by radiographic examinations and anatomic dissection of *Puma concolor*, as a flattened pyramid shape craniocaudally with a broad proximal base and rounded distal apex [11]. On CT, the patella was visualized as positioned within a symmetrical trochlear groove. Symmetry of the distal femur was also noted in lions (*Panthera leo*), suggesting a trend in cursorial carnivores [13]. HU values indicated density differences between the cortical and central bone of the patella. However, the cortical bone density of the patella was similar to the cortical bone of the other sesamoids.

The lateral (0.77 cm^2^) and medial (0.48 cm^2^) fabellae were easily visualized in all imaging methods, with the lateral fabella approximately 37.7% larger in size than the medial fabella, consistent with findings in other studies on *Puma concolor* [11,12]. In domestic cats, the lateral fabella is ossified and visible on radiographs, whereas the medial fabella is often not visualized, being in these cases histologically formed of fibrocartilage [8,14]. The popliteal sesamoid was most clearly visible in the mediolateral radiographic view and easily identifiable through CT reconstruction. It was the smallest of the sesamoids (0.22 cm^2^). In domestic cats, this sesamoid articulates with the lateral condyle of the tibia [15] and may fail to ossify [16]. A study of three *Puma concolor* specimens found that this sesamoid was embedded at the tendomuscular transition of the popliteus muscle [11].

The meniscus and cruciate ligaments were visualized on CT and MRI, but ultra-high-field MRI (7 Tesla) allowed these structures to be observed with precision. Following FDA (Food and Drug Administration) approval for clinical use in humans, 7 Tesla MRI has been used to diagnose meniscal injuries and changes in articular cartilage and subchondral bone due to its rapid image acquisition, high spatial resolution, and superiority in detecting early tissue changes [17,18]. No articular cartilage changes were observed in the stifle joint using DESS and FLASH sequences in the present study. Previous research in humans found similar sensitivity of FLASH and DESS sequences for longitudinal morphometry of stifle cartilage [19]. Additionally, the subchondral bone showed no changes when evaluated with the T2 sequence in the present study, which is considered the most accurate for detecting this type of injury [20].

In the stifle joints where meniscal mineralization was identified, it was recognized in all imaging modalities in the medial meniscus. A CT scan of *Panthera tigris* described the meniscal mineralization as dense cortical bone surrounding a less dense stroma, similar to the structure of the patella and fabella [10]. In the present study, meniscal mineralization exhibited a median HU of 971, which is lower than cortical bone (HU range of 1159.37–1363.5) and higher than trabecular bone (HU range of 373.87–632.12) found in various sesamoids.

The role of meniscal mineralization is always controversial in domestic and wild felines [10,21,22,23,24]. In wild felines, medial meniscal mineralization has been described in *Puma concolor*, *Panthera tigris*, *Acinonyx jubatus*, *Panthera leo*, *Panthera tigris*, *Panthera pardus*, and *Leopardus tigrinus*, but it was not associated with joint degenerative processes [10,11,21,23,25], as verified in the present study in the imaging analysis. Conversely, in domestic felines, one study attributed the presence of mineralization to degenerative joint disease [22], and another found that cats with a ruptured cranial cruciate ligament had a higher percentage of medium and large mineralizations compared to those without rupture [24].

In the present study, meniscal mineralization was detected in three adults and one young animal. The young animal had meniscal mineralization of a smaller size and a lower HU value than the adults. A study involving large felines suggested that meniscal ossicles mineralize with skeletal maturation and become radiographically visible around one year of age or in the last half of skeletal maturation [10]. The absence of meniscal mineralization in a young animal could be justified by this statement, but there were three adults in which mineralization was not identified, indicating that meniscal mineralization is not a constant finding. Furthermore, a study reported that *Panthera leo*, *Panthera tigris*, and *Panthera leo* with meniscal ossicles typically had a lateral fabella but often lacked the medial fabella of the gastrocnemius muscle [10]. This contrasts with the present study, where all animals had all sesamoids regardless of the presence or absence of mineralized medial meniscus.

To obtain a better understanding of meniscal mineralization, conducting studies that monitor animals in their natural habitat from birth and using periodic imaging exams to identify the timing of mineralization occurrence would be beneficial. Although meniscal mineralization in non-domestic felines may not be directly linked to stifle joint disease, it is important to consider that environmental factors could play a role in orthopedic diseases. This present study focused on pumas from a tropical region heavily impacted by human activities such as sugarcane plantations and increased road infrastructure, which limit the movement of these animals. Therefore, further research involving pumas from different regions is necessary to determine if environmental factors influence the development of meniscal mineralization.

A major limitation of this study was the small sample size of animals, which restricted the statistical analysis, especially regarding factors such as sex, age, and body mass. Further studies with a larger number of animals will be necessary. Moreover, imaging studies to assess growth plate closure in this species are essential to enhance the understanding of joint changes. However, the challenges in obtaining large felines for research purposes and the ethical concerns about the use of wild animals must be considered.

In conclusion, the descriptions of the stifle of *Puma concolor* in the different imaging methods contribute to understanding the species and can serve as a basis for identifying alterations.

## Figures and Tables

**Table 1:** Tomographic measurement areas (cm^2^) in the sagittal plane of the sesamoids and medial meniscus mineralization in the right and left stifle joints of eight pumas (*Puma concolor*).

No.	Age, Sex, Body Mass	Right Patella	Left Patella	Right Medial Fabella	Left Medial Fabella	Right Lateral Fabella	Left Lateral Fabella	Right Popliteal Sesamoid	Left Popliteal Sesamoid	Right Meniscus	Left Meniscus
1	Adult, M, 45.5 kg	3.18	2.96	0.7	0.62	0.98	1.06	0.28	0.27	0.02	0.08
2	Adult, M, 46.5 kg	2.68	2.37	0.57	0.55	1	1.06	0.25	0.28		
3	Adult, F, 37 kg	1.73	1.7	0.31	0.3	0.63	0.6	0.13	0.21	0.07	0.06
4	Adult, M, 45.5 kg	2.73	2.62	0.49	0.48	0.87	0.74	0.22	0.2		
5	Adult, F, 42 kg	2.2	2.09	0.44	0.43	0.72	0.68	0.2	0.19	0.07	0.09
6	Adult, F, 51 kg	2.87	3	0.54	0.49	0.44	0.82	0.24	0.24		
7	Young, F, 26.5 kg	2.56	2.3	0.29	0.31	0.67	0.58	0.17	0.15	0.01	0.01
8	Young, F, 36.5 kg	2.28	2.33	0.58	0.59	0.76	0.74	0.27	0.25		
	Mean ± SD	2.53 ± 0.45	2.42 ± 0.43	0.49 ± 0.14	0.47 ± 0.12	0.76 ± 0.19	0.78 ± 0.74	0.22 ± 0.05	0.22 ± 0.04	0.04 ± 0.03	0.06 ± 0.03

M = male, F = female.

**Table 2:** Hounsfield Unit values measured in sesamoids (compact bone—cortical and trabecular bone—central) and meniscus mineralization in the right stifle joints of eight pumas (*Puma concolor*).

No.	Age, Sex, Body Mass	Patella		Medial Fabella		Lateral Fabella		Popliteal Sesamoid		Meniscus
		Central	Cortical	Central	Cortical	Central	Cortical	Central	Cortical	
1	Adult, M, 45.5 kg	675	1227	362	1459	344	1559	614	1724	1335
2	Adult, M, 46.5 kg	662	1390	366	994	497	1174	713	938	
3	Adult, F, 37 kg	657	1206	420	1179	465	1448	706	1054	1099
4	Adult, M, 45.5 kg	640	1315	384	1468	446	1485	725	1426	
5	Adult, F, 42 kg	620	1078	331	1028	400	1211	544	1004	892
6	Adult, F, 51 kg	670	1164	352	801	365	1120	663	1001	
7	Young, F, 26.5 kg	551	1198	386	1236	425	1065	714	1226	398
8	Young, F, 36.5 kg	582	1145	390	1135	465	1355	300	902	
	Mean ± SD	632.12 ± 44.86	1215.37 ± 98.13	373.87 ± 27.14	1162.50 ± 228.29	425.87 ± 52.92	1302.12 ± 184.24	622.37 ± 144.38	1159.37 ± 285.33	931 ± 398.77

M = male, F = female.

**Table 3:** Hounsfield Unit values measured in sesamoids (compact bone—cortical and trabecular bone—central) and meniscus mineralization in the left stifle joints of eight pumas (*Puma concolor*).

No.	Age, Sex, Body Mass	Patella		Medial Fabella		Lateral Fabella		Popliteal Sesamoid		Meniscus
		Central	Cortical	Central	Cortical	Central	Cortical	Central	Cortical	
1	Adult, M, 45.5 kg	672	1383	450	1481	410	1749	676	1790	1354
2	Adult, M, 46.5 kg	658	1408	437	1022	506	1777	772	1095	
3	Adult, F, 37 kg	660	1208	412	1154	453	1528	659	1129	1050
4	Adult, M, 45.5 kg	647	1267	257	1376	358	1219	617	1408	
5	Adult, F, 42 kg	625	1080	409	1079	419	1158	524	971	878
6	Adult, F, 51 kg	508	1152	430	974	473	1118	647	1117	
7	Young, F, 26.5 kg	560	1165	377	1223	431	1123	780	1185	414
8	Young, F, 36.5 kg	573	1141	379	1227	444	1236	365	963	
	Mean ± SD	612.87 ± 50.09	1225.5 ± 118.03	393.87 ± 61.08	1192 ± 173.29	436.75 ± 44.22	1363.5 ± 278.81	630 ± 134.98	1207.25 ± 273.26	924 ± 392.85

M = male, F = female.

**Table 4:** Values of tomographic measurement areas (cm^2^) in the sagittal plane of the sesamoids and medial meniscus mineralization including all stifle joints of eight pumas (*Puma concolor*).

Mensuration Sites	Mean ± Standard Deviation	Minimum	Maximum	95% Confidence Interval
Patella	2.475 ± 0.431	1.700	3.180	2.246–2.704
Medial fabella	0.481 ± 0.126	0.290	0.700	0.4136–0.5476
Lateral fabella	0.772 ± 0.181	0.440	1.060	0.6752–0.8685
Popliteal sesamoid	0.222 ± 0.046	0.130	0.280	0.1972–0.2465
Medial meniscus	0.051 ± 0.033	0.010	0.090	0.02391–0.07859

**Table 5:** Hounsfield Unit values measured in sesamoids (compact bone—cortical and trabecular bone—central) and meniscus mineralization including all stifle joints of eight pumas (*Puma concolor*).

Mensuration Sites		Median	Standard Error	Minimum	Maximum	95% Confidence Interval	1°–3° Quartile
Patella	Central	643.50	12.91	508.00	675.00	595.0–650	575.3–661.5
	Cortical	1202.00	26.25	1078.00	1408.00	1164–1276	1147–1303
Medial fabella	Central	385.00	11.70	257.00	450.00	358.9–408.8	363.0–418.0
	Cortical	1167.00	49.10	801.00	1481.00	1073–1282	1024–1341
Lateral fabella	Central	437.50	11.86	344.00	506.00	406.0–456.6	402.5–465.0
	Cortical	1228.00	57.62	1065.00	1777.00	1210–1456	1132–1777
Popliteal sesamoid	Central	661.00	33.77	300.00	780.00	554.2–698.2	561.5–780.0
	Cortical	1106.00	67.76	902.00	1790.00	1039–1328	978.5–1790
Medial meniscus		971.00	129.60	398.00	1354.00	621.1–1234	530.0–1354

**Table 6:** Comparison between Hounsfield Units (trabecular bone—central and compact bone—cortical) of the patella, fabella, and popliteal sesamoid measured including all stifle joints of eight pumas (*Puma concolor*).

Variables	*p* Values	Statistical Test
Central patella X Cortical patella	<0.0001	Paired T
Central patella X Central fabella	<0.0001	Paired T
Central patella X Cortical fabella	0.0005	Wilcoxon
Central patella X Central popliteal sesamoid	0.8160	Wilcoxon
Central patella X Cortical popliteal sesamoid	<0.0001	Paired T
Cortical patella X Central fabella	<0.0001	Paired T
Cortical patella X Cortical fabella	0.7761	Wilcoxon
Cortical patella X Central popliteal sesamoid	<0.0001	Wilcoxon
Cortical patella X Cortical popliteal sesamoid	0.5516	Paired T

## Data Availability

The data presented in this study are available on request from the corresponding author.
